# Low-grade proteinuria and atherosclerotic cardiovascular disease: A transition study of patients with diabetic kidney disease

**DOI:** 10.1371/journal.pone.0264568

**Published:** 2022-02-25

**Authors:** Satoshi Yamaguchi, Takayuki Hamano, Tatsufumi Oka, Yohei Doi, Sachio Kajimoto, Yusuke Sakaguchi, Akira Suzuki, Yoshitaka Isaka

**Affiliations:** 1 Department of Nephrology, Osaka University Graduate School of Medicine, Suita, Osaka, Japan; 2 Department of Internal Medicine, Japan Community Health Care Organization Osaka Hospital, Osaka, Osaka, Japan; 3 Department of Nephrology, Nagoya City University Graduate School of Medical Sciences, Nagoya, Aichi, Japan; 4 Department of Inter-Organ Communication Research in Kidney Disease, Osaka University Graduate School of Medicine, Suita, Osaka, Japan; University of Sao Paulo Medical School, BRAZIL

## Abstract

Diabetic kidney disease (DKD) is heterogeneous in terms of proteinuria. Patients with DKD who present with low-grade proteinuria are more likely to have nephrosclerosis rather than traditional diabetic nephropathy. The amount of proteinuria might reflect the underlying pathology of renal failure and influence the prognosis after dialysis initiation. Clinical implications of proteinuria at the start of dialysis have not been confirmed, while greater proteinuria is associated with higher risk of cardiovascular disease (CVD) in the predialysis stages of chronic kidney disease. We performed a retrospective multicenter cohort study enrolling incident hemodialysis patients with diabetes. Patients were stratified using proteinuria quartiles. We examined the association of proteinuria quartiles with types of subsequent CVD. Among the enrolled 361 patients, the estimated mean glomerular filtration rate and proteinuria was 5.4 mL/min/1.73 m^2^ and 6.3 g/gCr, respectively. Lower quartile of proteinuria (cut-offs: 3.0, 5.4, and 8.8 g/gCr) was significantly associated with male, older age, and history of atherosclerotic CVD including coronary artery disease, peripheral arterial disease, and cerebral infarction (P_trend_<0.05). Kidney size was smaller in patients with lower levels of proteinuria. Patients with higher levels of proteinuria were more likely to have proliferative diabetic retinopathy (P_trend_<0.05). Multivariate competing risk analysis revealed that the first quartile of proteinuria was associated with a greater risk of atherosclerotic CVD than the third quartile (subhazard ratio [95% confidence interval]: 2.04 [1.00–4.14]). This association was attenuated after additional adjustments for history of atherosclerotic CVD. Furthermore, patients with lower quartiles of proteinuria were more likely to die of atherosclerotic CVD than those with non-atherosclerotic CVD (P_trend_ = 0.01). Diabetic patients with lower proteinuria at dialysis initiation were characterized by severer macroangiopathy, as shown by a more atrophic kidney and higher prevalence of past atherosclerotic CVD. Hence, they are at a high risk of developing atherosclerotic CVD.

## Introduction

Diabetic kidney disease is heterogeneous: patients with diabetes develop kidney disease due to diabetes per se and/or other comorbidities, including aging-related nephron loss and hypertension [1]. Among patients with classical diabetic nephropathy, macroalbuminuria usually precedes the decline of renal function (proteinuric pathway) [2, 3]. In contrast, some patients with diabetes develop renal insufficiency without albuminuria [4], similar to patients with nephrosclerosis. In fact, a previous study showed that most patients with diabetic nephropathy and estimated glomerular filtration rate (eGFR)<60 mL/min/1.73 m^2^ had concomitant macroalbuminuria, while a small proportion of patients with nephrosclerosis showed macroalbuminuria even when their eGFR reached 45 mL/min/1.73 m^2^ [5]. Furthermore, the population-based National Health and Nutrition Examination Survey showed that the prevalence of albuminuria (albuminuria-to-creatinine ratio ≥30 mg/g) decreased progressively from 20.8% in 1988–1994 to 15.9% in 2009–2014, while the prevalence of reduced eGFR (<60 mL/min/1.73 m^2^) increased from 9.2% in 1988–1994 to 14.1% in 2009–2014 [6]. In patients with diabetes, non-proteinuric pathways, which include obesity, dyslipidemia, and hypertension, are also involved in the loss of renal function [3].

These non-proteinuric pathway components are risk factors for atherosclerosis [7, 8]. In a study enrolling patients with biopsy-proven diabetic nephropathy, patients with normoalbuminuria and eGFR<60 mL/min/1.73 m^2^ were reported to have pathologically more severe atherosclerosis (intimal thickening) in the kidney than patients with microalbuminuria or macroalbuminuria and eGFR<60 mL/min/1.73 m^2^ [9]. Intimal thickening suggests the involvement of hypertension [10]. In this context, non-proteinuric pathways may have contributed to atherosclerosis in patients with diabetes. Moreover, these patients with diabetes and low levels of proteinuria at dialysis initiation might have suffered from a more severe atherosclerosis during pre-dialysis chronic kidney disease (CKD). The deterioration of renal function might be attributed to non-proteinuric pathways rather than the proteinuric pathway. In addition, patients with diabetes and modest proteinuria might have smaller kidneys than patients with severe proteinuria, because atherosclerosis, accompanied with aging, accelerates kidney atrophy [11]. Supposing that moderate proteinuria at dialysis initiation reflects the severity of atherosclerosis, it might also predict future atherosclerotic cardiovascular disease after dialysis initiation. Regarding the prognostic implications of proteinuria, a previous study including 105,872 participants showed that the albumin/creatinine ratio was linearly associated with the risk of cardiovascular mortality on a log-log scale without threshold effects [12]. However, in this study, patients with eGFR < 15 mL/min/1.73 m^2^ accounted for only 0.1% of all participants [12]. Therefore, it remains to be elucidated whether proteinuria at dialysis initiation is useful for risk stratification.

Therefore, we examined the association between the amount of proteinuria and prior history of atherosclerotic cardiovascular disease (CVD), classical diabetic nephropathy, and renal size among incident dialysis patients. We also examined the association between the amount of proteinuria and subsequent CVD after dialysis initiation.

## Materials and methods

### Study design and populations

In this retrospective multicenter cohort study, we enrolled diabetic patients with urinary protein-to-creatinine ratio (UPCR) measured who were introduced to hemodialysis between January 2008 and December 2016 at the Osaka University Hospital or between June 2008 and July 2018 at the Japan Community Health care Organization (JCHO) Osaka Hospital. Patients who started dialysis at the intensive care unit of Osaka University Hospital were excluded. The Ethics Committee of Osaka University Hospital approved this study and waived the need for informed consent due to its retrospective study design (approval number: 18026). Patients were provided with the option to opt out of participation. The study was conducted in accordance with the Declaration of Helsinki.

### Data collection

We used the latest data collected within 3 months prior to dialysis initiation. For the analysis of renal size, we collected pole-to-pole kidney length measured between 6 months before and 1 month after dialysis initiation. In patients with multiple data during the study period, we selected the nearest data in relation to the timing of dialysis initiation. Kidney length was measured using 3D-computed tomography (CT) images created by the Synapse Vincent System (FUJIFILM Corporation, Tokyo, Japan). For patients without CT data, kidney length was measured using ultrasonography. We recorded body weights just before the first dialysis (BW) and dry weights (DW) just before hospital discharge. We defined the overhydration rate (OH-R), reflecting volume overload at dialysis initiation, using the following formula: OH-R (%) = [(BW-DW)/DW] × 100.

### Outcomes

We followed up patients who initiated dialysis at Osaka University Hospital and JCHO Osaka Hospital through August 2017 and September 2018, respectively. All patients started dialysis after admission to the hospital in our cohort. The primary outcome was fatal atherosclerotic CVD and re-hospitalization for atherosclerotic CVD. Atherosclerotic CVD consists of myocardial infarction, unstable angina, non-hemorrhagic stroke, peripheral vascular disease (including amputation), and aortic dissection or rupture. The rest of the CVD cases were non-atherosclerotic CVD, which includes heart failure, arrhythmia, hemorrhagic stroke, sudden death, fatal pulmonary embolism, and death due to unspecified causes of CVD. The outcomes were obtained from medical records or common questionnaires sent to each dialysis facility. Patients were censored at the date of kidney transplantation or when they were lost to follow-up.

### Statistical analyses

Patients were categorized into quartiles of UPCR, with cut-offs at 3.0, 5.4, and 8.8 g/gCr. For continuous variables, the mean (standard deviation) was presented, while percentages were presented for categorical variables. We examined linear trends of baseline characteristics across quartiles of UPCR using linear or logistic regression, as appropriate. To explore a potentially non-linear relationship between UPCR and renal length, we adopted restricted cubic spline models adjusted for age, sex, eGFR, height, body mass index at discharge, and modality for measurement of renal length (CT or ultrasonography).

We examined the association between quartiles of UPCR and first atherosclerotic CVD events using competing risk regression models with death from diseases other than atherosclerotic CVD as the competing risk. After conducting a univariate analysis, we constructed several multivariate models: model 1 adjusted for age, sex, eGFR, body mass index at discharge, systolic blood pressure, glycated hemoglobin A1c (HbA1c), smoking status, and prescription (aspirin, statin, and insulin); and model 2 adjusted for covariates in model 1 plus history of atherosclerotic CVD (percutaneous coronary intervention or coronary artery bypass grafting, peripheral arterial disease, and cerebral infarction). Additionally, we examined the association between quartiles of UPCR and fatal atherosclerotic CVD using competing risk regression models with death from other diseases than atherosclerotic CVD as the competing risk.

Statistical significance was set at P < 0.05. All statistical analyses were performed using Stata/IC 14 (StataCorp, College Station, TX, USA).

## Results

### Baseline characteristics

**Fig 1:**
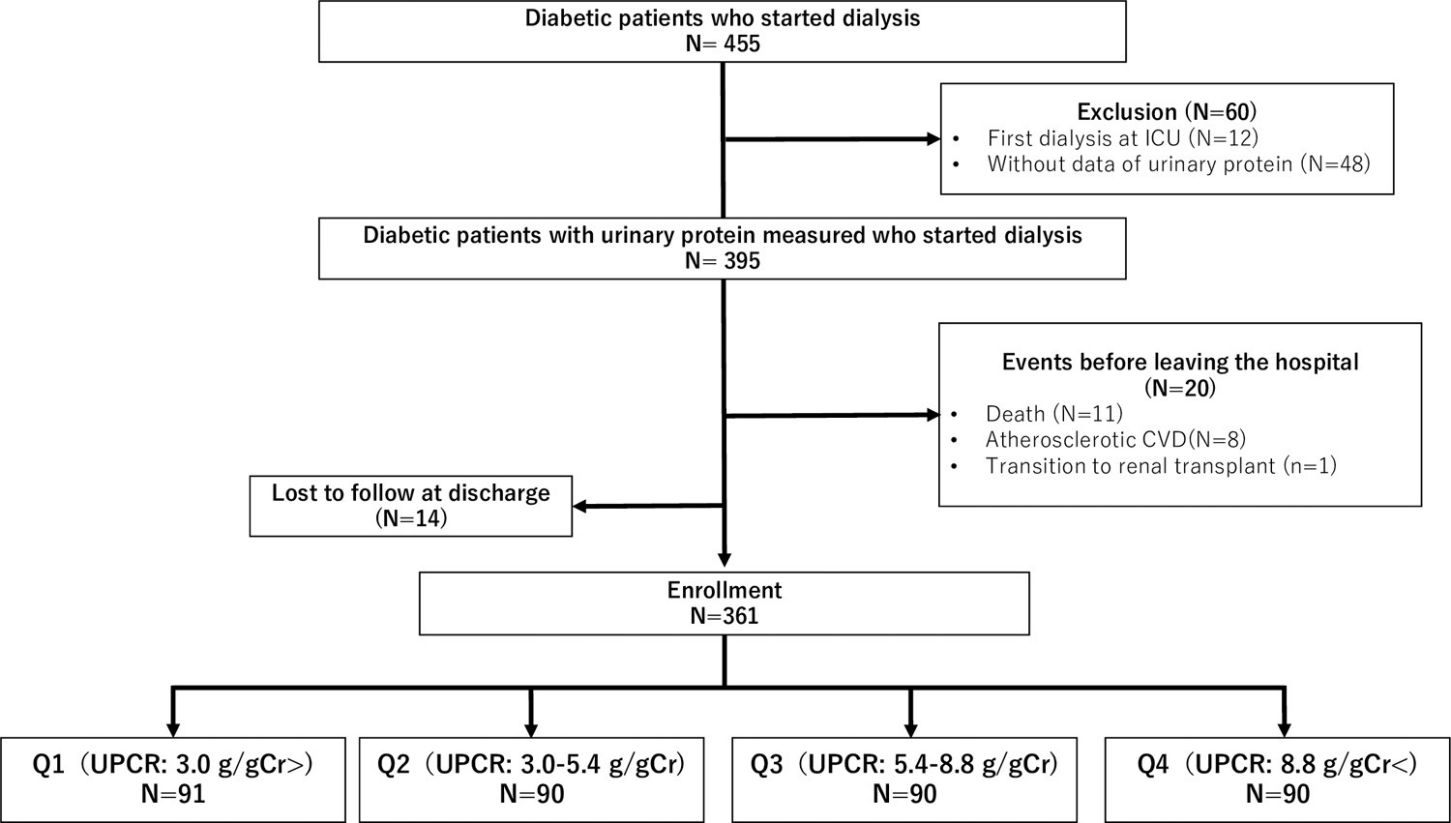
Flow diagram. Abbreviations: CVD, cardiovascular disease; ICU, intensive care unit; Q1-4, quartile 1–4; UPCR, urine protein-to-creatinine ratio.

**Fig 2:**
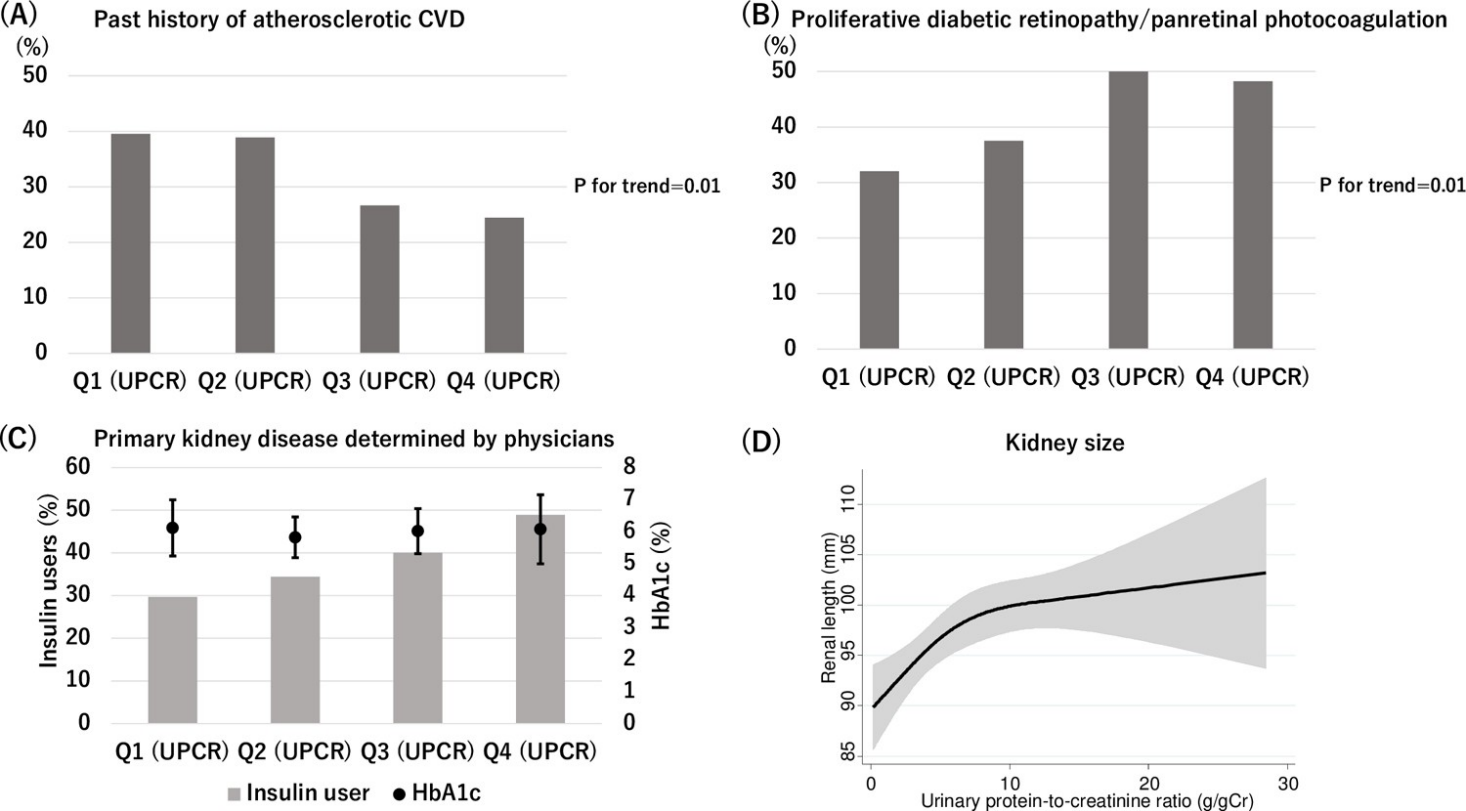
Baseline characteristics across the quartiles of UPCR. (A) Patients with lower quartile of UPCR are more likely to have a history of atherosclerotic CVD. (B) Patients with greater quartile of UPCR are more likely to have advanced diabetic retinopathy (proliferative diabetic retinopathy or history of panretinal photocoagulation). (C) Patients with greater quartile of proteinuria tend to receive insulin at dialysis initiation (P for trend = 0.01). There are no significant differences in HbA1c levels across UPCR quartiles. (D) Patients with low levels of proteinuria have small renal length. The association is adjusted for age, sex, eGFR, height, body mass index at discharge, and modality for measurement of renal length (CT or ultrasonography). Abbreviations: CVD, cardiovascular disease; eGFR, estimated glomerular filtration rate; HbA1c, glycated hemoglobin A1c.

In our cohort study, urinary protein levels were measured in 89% of patients with diabetes who started dialysis (Fig 1). There was no significant difference in the variables predicting subsequent atherosclerotic CVD between patients with and without the data of urinary protein (S1 Table). Among the 361 enrolled patients (Fig 1), the mean age was 66 years, 73% were male, and the mean eGFR was 5.4 mL/min/1.73 m^2^. Patients in the lower quartile of UPCR were more likely to be male and elderly. They were more likely to have a history of atherosclerotic CVD, including coronary artery disease, peripheral arterial disease, and cerebral infarction (Fig 2A). There was a significant tendency toward higher OH-R and systolic blood pressure with the increase in UPCR, suggesting overhydration in patients with higher UPCR. Patients with massive proteinuria had lower albumin levels accompanied with higher low-density lipoprotein (LDL) levels despite having a higher likelihood of receiving statins. They were more likely to have preserved renal length (Table 1). This association was observed after adjustment for variables including age, sex, eGFR, height, and body mass index (Fig 2D). Patients with higher levels of proteinuria were more likely to be diagnosed with proliferative diabetic retinopathy or had received panretinal photocoagulation (P for trend<0.05) (Fig 2B). Although the duration of diabetes and HbA1c levels were similar across the quartiles of UPCR, patients with higher levels of proteinuria were more likely to receive a prescription of insulin at the start of dialysis (Fig 2C).

**Table 1:** Baseline characteristics.

Variables	UPCR				P for trend
	Q1 (< 3.0 g/gCr)	Q2 (3.0–5.4 g/gCr)	Q3 (5.4–8.8 g/gCr)	Q4(> 8.8 g/gCr)	
	N = 91	N = 90	N = 91	N = 90	
UPCR (g/gCr)	1.9 [1.2–2.5]	4.1 [3.6–4.6]	6.7 [6.1–7.6]	11.3 [9.9–14.1]	<0.001
Age (years)	69.0 (11.4)	67.6 (11.4)	64.1 (13.9)	61.5 (13.4)	<0.001
Male (%)	82.4	74.4	65.6	68.9	0.02
Dialysis catheters (%)	8.8	2.2	6.7	2.2	0.14
Classification of diabetes					
Type 1 (%)	2.2	2.2	6.7	3.3	0.37
Type 2 (%)	96.7	97.8	93.3	96.7	0.62
Pancreatic (%)	1.1	0	0	0	NA
Diabetes duration (years)	15.5 [7.5–26.0]	14.6 [9.2–22.4]	15.3 [8.4–20.7]	14.9 [8.0–22.0]	0.67
Systolic blood pressure (mmHg)	145 (25)	149 (21)	155 (24)	161 (25)	<0.001
eGFR (mL/min/1.73m^2^)	6.1 (2.5)	5.2 (1.7)	5.2 (1.7)	4.9 (1.9)	<0.001
Renal length (mm)	94.2 (12.9)	93.7 (13.0)	99.1 (13.5)	102.1 (12.8)	<0.001
Body mass index					
At dialysis initiation	24.2 (4.2)	25.0 (4.4)	24.6 (4.1)	26.9 (5.4)	0.001
At discharge	22.5 (3.9)	23.0 (3.8)	22.3 (3.9)	23.1 (3.9)	0.49
Overhydration rate (%)	6.3 [2.3–11.8]	6.4 [3.9–10.8]	8.7 [3.6–16.4]	14.6 [6.9–22.8]	<0.001
Laboratory data					
Albumin (g/dL)	3.3 (0.6)	3.1 (0.5)	2.9 (0.5)	2.5 (0.5)	<0.001
LDL (mg/dL)	79 (24)	87 (30)	93 (35)	109 (52)	<0.001
HbA1c (%)	6.1 (0.9)	5.8 (0.6)	6.0 (0.7)	6.1 (1.1)	0.85
Prior history (%)					
Advanced diabetic retinopathy	32.1	37.5	53.2	48.2	0.01
Coronary artery disease	24.2	17.8	12.2	12.2	0.02
Heart failure	30.8	25.6	20.0	13.3	0.004
Cerebral infarction	15.4	18.9	11.1	5.6	0.02
Peripheral arterial disease	15.4	13.3	5.6	11.1	0.17
Smoking status (%)					
Current	18.7	12.2	21.1	30.0	0.03
Past	30.8	27.8	20.0	28.9	0.52
Never	44.0	56.7	55.6	40.0	0.59
Unknown	6.6	3.3	3.3	1.1	0.07
Prescriptions (%)					
Aspirin	39.6	43.3	34.4	30.0	0.10
Statin	29.7	41.1	45.6	54.4	0.001
Insulin	29.7	34.4	40.0	48.9	0.01
RAAS inhibitors	58.2	56.7	52.2	51.1	0.27

Data are presented as mean standard deviation (SD), median (interquartile range), or percentages. P for trend across quartiles of UPCR is examined using linear or logistic regression, as appropriate. Advanced diabetic retinopathy is defined as proliferative diabetic retinopathy or a history of panretinal photocoagulation. Log (OH-R+6) is used for the logarithmic transformation of the overhydration rate.

Abbreviations: eGFR, estimated glomerular filtration rate; HbA1c, glycated hemoglobin A1c; LDL, low density lipoprotein; NA, not available; OH-R, overhydration rate; RAAS, renin-angiotensin-aldosterone system; UPCR, urinary protein creatinine ratio.

### Future atherosclerotic CVD

**Fig 3:**
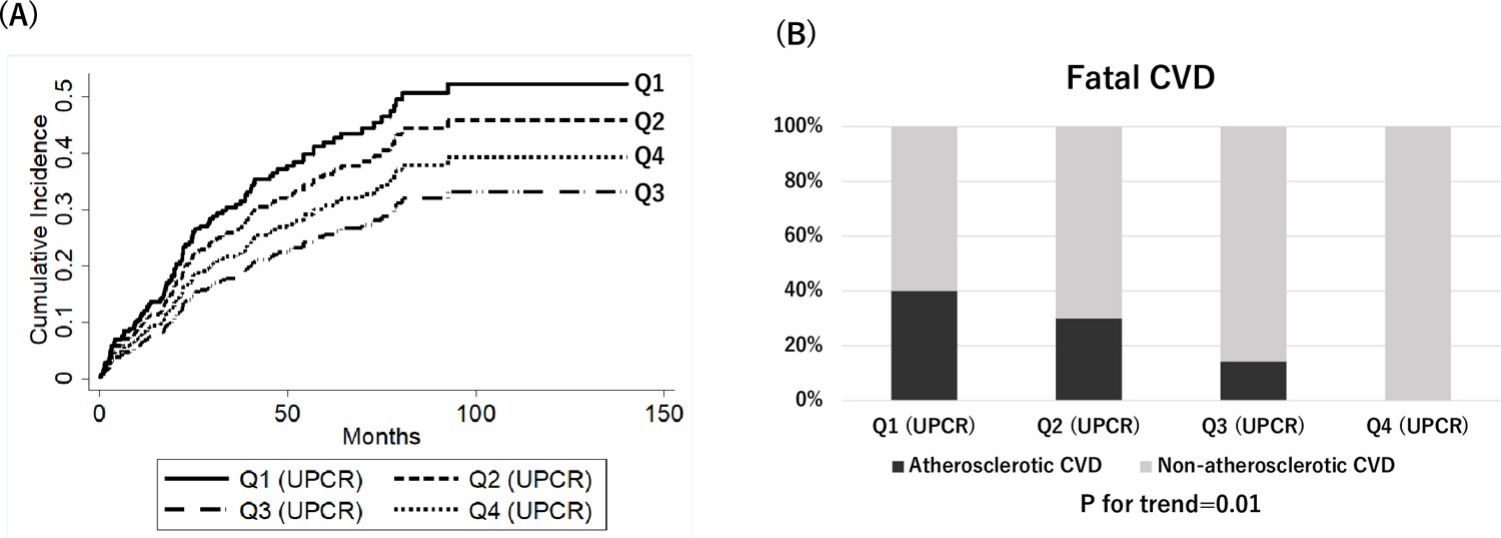
Cumulative incidence of atherosclerotic CVD after dialysis initiation (A) and percentages of atherosclerotic CVD to total fatal CVD by UPCR quartile. Patients in UPCR Q1 have a significantly higher risk for atherosclerotic CVD than patients in UPCR Q3 (subhazard ratio [95% confidence interval]: 1.84 [1.06–3.17]). Abbreviations: CVD, cardiovascular disease; Q1-4, quartiles 1–4; UPCR, urine protein-to-creatinine ratio.

During a median follow-up period of 36 months (IQR, 19–68), 103 patients (28.5%) developed atherosclerotic CVD. In univariate competing risk analysis, the first quartile (Q1) of UPCR was a significant risk factor for atherosclerotic CVD (subhazard ratio (SHR) [95% confidence interval (CI)]: 1.84 [1.06–3.17] compared to quartile 3) (Table 2) (Fig 3A). After adjustment for variables including age, sex, eGFR, body mass index at discharge, systolic blood pressure, HbA1c, prescription, and smoking status, UPCR Q1 remained a significant risk factor for atherosclerotic CVD (SHR [95%CI]: 2.04 [1.00–4.14]). This association was attenuated after additional adjustment for history of atherosclerotic CVD (SHR [95%CI]: 1.58 [0.78–3.22]). Furthermore, patients with lower quartiles of proteinuria were more likely to die of atherosclerotic CVD using competing risk regression analysis (P for trend = 0.01) (Fig 3B).

**Table 2:** Association of quartile with atherosclerotic CVD.

	Univariate		Model 1		Model 2	
	Subhazard ratio [95%CI]	P value	Subhazard ratio [95%CI]	P value	Subhazard ratio [95%CI]	P value
Q1 (UPCR)	1.84 [1.06–3.17]	0.03	2.04 [1.00–4.14]	0.049	1.58 [0.78–3.22]	0.21
Q2 (UPCR)	1.53 [0.87–2.67]	0.14	1.69 [0.89–3.23]	0.11	1.50 [0.80–2.80]	0.20
Q3 (UPCR)	Reference		Reference		Reference	
Q4 (UPCR)	1.24 [0.68–2.25]	0.48	1.29 [0.65–2.58]	0.46	1.27 [0.65–2.48]	0.49

Model 1: univariate + age, sex, eGFR, body mass index, systolic blood pressure, HbA1c, smoking status, and prescription (aspirin, statin, and insulin).

Model 2: Model 1 + history of atherosclerotic CVD (percutaneous coronary intervention or coronary artery bypass grafting, peripheral arterial disease, and cerebral infarction)

Abbreviations: CVD, cardiovascular disease; eGFR, estimated glomerular filtration rate; HbA1c, glycated hemoglobin A1c; Q1-4, quartiles 1–4; UPCR, urinary protein creatinine ratio

## Discussion

**Fig 4:**
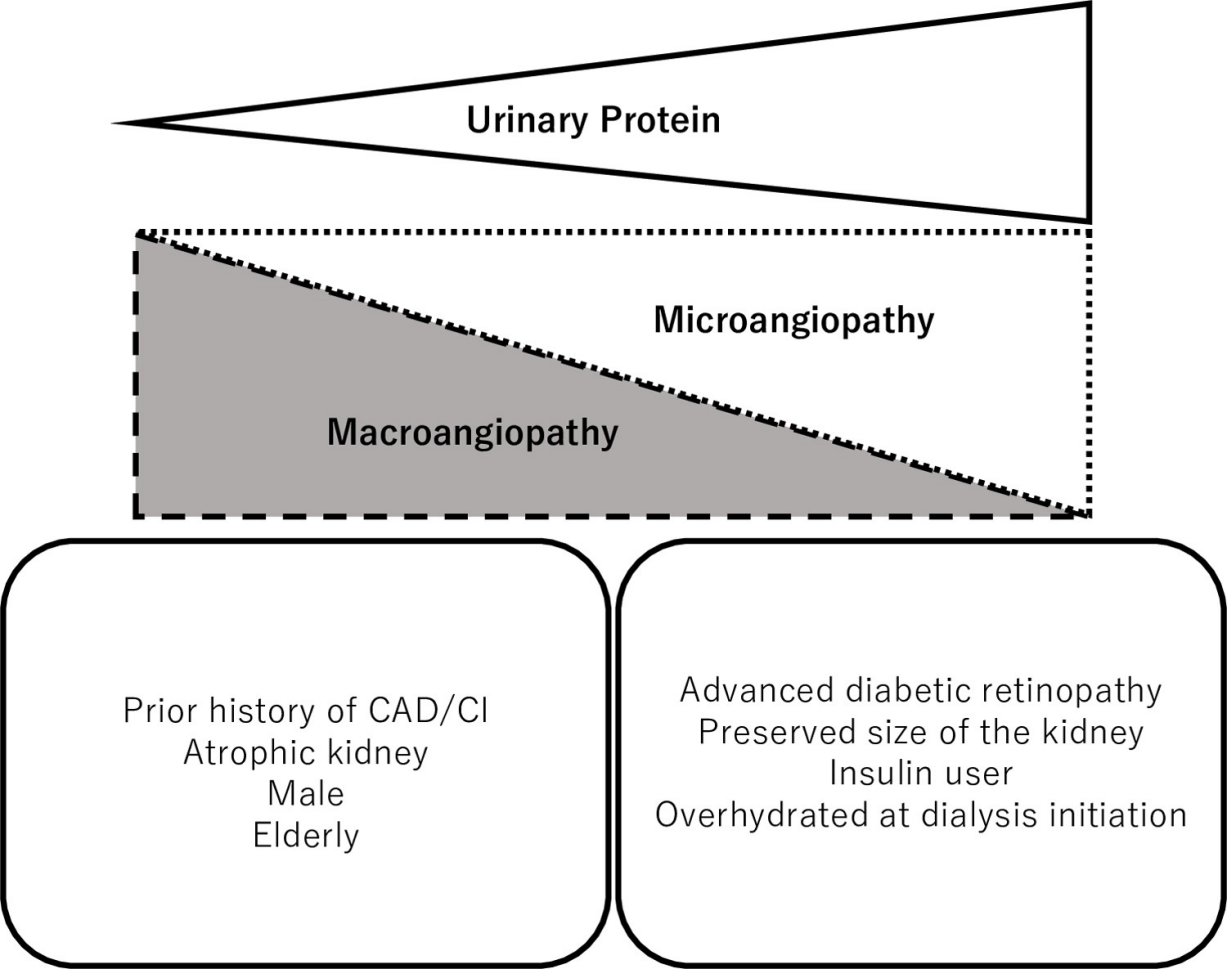
Heterogeneity of DKD pathogenesis and amount of proteinuria: Dominancy of macroangiopathy or microangiopathy. Patients with modest proteinuria are more likely to be male and elderly. They tend to have an atrophic kidney and a history of atherosclerotic CVD (macroangiopathy). Meanwhile, patients with massive proteinuria tend to receive insulin and suffer from overhydration. They are characterized by preserved kidney size and advanced diabetic retinopathy (microangiopathy). Abbreviations: CAD, coronary artery disease; CI, cerebral infarction; DKD, diabetic kidney disease.

In this multicenter retrospective cohort study enrolling incident dialysis patients with diabetes, we showed that patients with severe proteinuria were more likely to have advanced diabetic retinopathy (microangiopathy) and to receive insulin at dialysis initiation. In contrast, patients with moderate proteinuria were more likely to have atrophic kidneys and a history of atherosclerotic CVD (macroangiopathy) (Fig 4). Furthermore, low proteinuria at dialysis initiation was associated with subsequent atherosclerotic CVD.

Patients with more severe proteinuria had lower serum albumin and higher LDL levels. In other words, patients with massive proteinuria showed nephrotic syndrome. They were more likely to receive statins, probably because of their high LDL levels. Furthermore, these patients also had lower eGFR at dialysis initiation and higher OH-R. They might have withstood more severe overhydration without suffering from congestive heart failure. This is probably because edema rather than increased intravascular volume contributed to overhydration since they have low serum osmotic pressure accompanied with vascular hyperpermeability, as it is often observed in patients with diabetes [13]. In addition, patients with massive proteinuria tended to have preserved renal size, which is characteristic of classical diabetic nephropathy [14].

Patients with massive proteinuria were more likely to receive insulin and have advanced diabetic retinopathy. Although we cannot prove causality in our study, insulin therapy was unlikely to cause microangiopathy because multiple insulin injection treatments (three times per day) are more effective to prevent the progression of microangiopathy than conventional insulin injection therapy (one to two times per day) [15]. Furthermore, intensive blood glucose control, either by oral antidiabetic agents or insulin, has been shown to reduce the risk of microangiopathy [16, 17]. Meanwhile, in previous studies, intensive blood glucose control did not reduce the risk of macroangiopathy in early time course of glycemic control [16–18]. In fact, HbA1c levels ≥ 7.0% were reported to be associated with microvascular complications, including retinopathy, but not macrovascular complications such as coronary artery disease, cerebral infarction, and peripheral arterial disease [19]. These findings suggest a stronger association between blood glucose levels and microangiopathy than with macroangiopathy. Taken together, patients with severe proteinuria at dialysis initiation might have been exposed to hyperglycemia refractory to oral antidiabetic agents before the start of insulin therapy, resulting in the progression of microangiopathy.

Another possibility is that the necessity of insulin prescription at dialysis initiation might reflect severely decreased insulin secretion due to β-cell dysfunction, which was caused by islet microangiopathy driven by long-term hyperglycemia. In db/db mice, signs of islet microangiopathy have been reported. The observed findings include increase in the average and diversity of islet capillary size, pericapillary edema and fibrosis, and hypertrophy of the pericyte with abundant actin-like microfilaments, which suggest capillary hyperperfusion, hypertension, and secondary vascular response [20]. Hemodynamic changes and vascular responses are considered important components of diabetic microangiopathy [21]. Our findings are compatible with those of a previous cross-sectional study, which showed an association between the presence of microangiopathy and β-cell dysfunction [22].

Patients with modest proteinuria were more likely to have a history of atherosclerotic CVD. In addition, they were more likely to be elderly and male, which have been reported as risk factors for atherosclerosis [23, 24]. Patients with lower proteinuria had more atrophic kidneys, suggesting that nephrosclerosis or ischemic nephropathy caused by renal artery sclerosis could have contributed to the deterioration of renal function. Ischemic nephropathy is characterized by decreased glomerular pressure, which partially explains the relatively low proteinuria levels since nephrosclerosis or renal artery sclerosis is a manifestation of advanced atherosclerosis in the whole body. The observed association between modest proteinuria and future atherosclerotic events is reasonable.

It should be noted that more than 95% of patients in our study had proteinuria corresponding to A3 levels of albuminuria (>300 mg/g) according to the Kidney Disease: Improving Global Outcomes (KDIGO) criteria. Our findings indicate that the link between severe proteinuria and worsening prognosis advocated by the KDIGO guidelines cannot be extrapolated to those patients with end-stage renal disease who are new to dialysis. Proteinuria accelerates kidney function loss [25] that has a strong association with CVD [26]. In other words, proteinuria is associated with subsequent CVD through renal function loss among patients with pre-dialysis patients with CKD [27]. After hemodialysis initiation, residual renal function deteriorates rapidly [28], which possibly diminishes the association between proteinuria and CVD risk on maintenance hemodialysis.

Few studies have investigated the association between proteinuria at the initiation of dialysis and prognosis. The findings of our study are consistent with those of previous studies that enrolled patients with advanced CKD. In pre-dialysis patients with eGFR < 30 mL/min/1.73 m^2^, a J-shaped association between proteinuria and all-cause mortality has been reported [12, 29]. Among incident dialysis patients, pre-dialysis proteinuria was reported to be inversely associated with all-cause mortality [30]. In our study, patients with lower proteinuria were more likely to have a history of coronary artery disease and cerebral infarction. Additional adjustment for the history of atherosclerotic CVD, such as cerebral infarction and coronary artery disease, attenuated the association of low proteinuria with the risk of subsequent atherosclerotic CVD. Furthermore, the lower the amount of proteinuria, the higher percentage of atherosclerotic CVD among patients who died of any CVD. These data suggest that atherosclerosis developed during pre-dialysis CKD contributes to the risk of atherosclerotic CVD events after the initiation of dialysis.

Patients in the fourth quartile of proteinuria had a greater risk of future atherosclerotic CVD than those in the third quartile of proteinuria. This might be explained by long-term effects of glycemic control. Patients in the fourth quartile of proteinuria might have had poor glycemic control as shown in the high prevalence of insulin users. In the UK Prospective Diabetes Study, intensive glucose control decreases the risk of microvascular complications, but not macrovascular complications during the first 10 years after diagnose of diabetes [16]. However, a risk reduction for myocardial infarction was observed during the additional follow-up of 10 years after the trial [31]. Besides, patients in the fourth quartile of proteinuria had a significantly higher LDL level than patients in the third quartile (109 versus 93, P = 0.02). Since high LDL levels are risk factors for future atherosclerotic CVD [32], this significant difference in LDL levels might explain a greater risk of atherosclerotic CVD in the fourth quartile of proteinuria.

This study had several strengths. First, this is the first study to examine the association between the amount of proteinuria at the initiation of dialysis and the characteristics of patients with diabetes. Second, we recorded kidney length at the initiation of dialysis. No study has examined the association between the amount of proteinuria and kidney size in incident dialysis patients. In contrast, this study also had several limitations. First, the outcomes were not adjudicated. The indications for hospitalization might vary among physicians. Second, we enrolled only patients with diabetes, so our results cannot be extrapolated to patients without diabetes. Third, the method of measuring renal length was not standardized among the technicians.

In conclusion, patients with diabetes and with lower levels of proteinuria at dialysis initiation were more likely to have prevalent atherosclerotic CVD at the initiation of dialysis and a higher risk of developing atherosclerotic CVD after the initiation of dialysis. The amount of proteinuria at dialysis initiation might be useful in determining the heterogeneous pathologies of patients with diabetes: the dominance of microangiopathy and macroangiopathy.

## Supporting information

S1 Table Characteristics of patients with or without the data of UPCR. Abbreviations: eGFR, estimated glomerular filtration rate; HbA1c, glycated hemoglobin A1c; LDL, low density lipoprotein; OH-R, overhydration rate; RAAS, renin-angiotensin-aldosterone system; UPCR, urinary protein creatinine ratio.
(PDF)

## References

[1] Thomas MC, Brownlee M, Susztak K, Sharma K, Jandeleit-Dahm KA, Zoungas S, et al.: Diabetic kidney disease. Nat Rev Dis Primers 2015;30(1):15018. doi: 10.1038/nrdp.2015.18 27188921 PMC7724636

[2] Pugliese G: Updating the natural history of diabetic nephropathy. Acta Diabetol. 2014;51(6):905–15. doi: 10.1007/s00592-014-0650-7 25297841

[3] Porrini E, Ruggenenti P, Mogensen CE, Barlovic DP, Praga M, Cruzado JM, et al.; ERA-EDTA diabesity working group: Non-proteinuric pathways in loss of renal function in patients with type 2 diabetes. Lancet Diabetes Endocrinol. 2015;3(5):382–91. doi: 10.1016/S2213-8587(15)00094-7 25943757

[4] Molitch ME, Steffes M, Sun W, Rutledge B, Cleary P, de Boer IH, et al.; Epidemiology of Diabetes Interventions and Complications Study Group: Development and progression of renal insufficiency with and without albuminuria in adults with type1 diabetes in the diabetes control and complications trial and the epidemiology of diabetes interventions and complications study. Diabetes Care. 2010;33(7):1536–43. doi: 10.2337/dc09-1098 20413518 PMC2890355

[5] Abe M, Okada K, Maruyama N, Takashima H, Oikawa O, Soma M: Comparison of Clinical Trajectories before Initiation of Renal Replacement Therapy between Diabetic Nephropathy and Nephrosclerosis on the KDIGO Guidelines Heat Map. J Diabetes Res. 2016;2016:5374746. doi: 10.1155/2016/5374746 26839894 PMC4709677

[6] Afkarian M, Zelnick LR, Hall YN, Heagerty PJ, Tuttle K, Weiss NS, et al.: Clinical manifestations of kidney disease among US adults with diabetes, 1988–2014. JAMA. 2016;316(6):602–10. doi: 10.1001/jama.2016.10924 27532915 PMC5444809

[7] Alexander RW: Theodore Cooper Memorial Lecture. Hypertension and the pathogenesis of atherosclerosis. Oxidative stress and the mediation of arterial inflammatory response: a new perspective. Hypertension. 1995;25(2):155–61. doi: 10.1161/01.hyp.25.2.155 7843763

[8] Yoo HJ, Choi KM: Adipokines as a novel link between obesity and atherosclerosis. World J Diabetes. 2014;5(3):357–63. doi: 10.4239/wjd.v5.i3.357 24936256 PMC4058739

[9] Shimizu M, Furuichi K, Toyama T, Kitajima S, Hara A, Kitagawa K, et al.: Kanazawa Study Group for Renal Diseases and Hypertension: Long-term outcomes of Japanese type 2 diabetic patients with biopsy-proven diabetic nephropathy. Diabetes Care. 2013;36(11):3655–62. doi: 10.2337/dc13-0298 24089538 PMC3816871

[10] Freedman BI, Iskandar SS, Appel RG: The link between hypertension and nephrosclerosis. Am J Kidney Dis. 1995;25(2):207–21. doi: 10.1016/0272-6386(95)90001-2 7847347

[11] Bax L, van der Graaf Y, Rabelink AJ, Algra A, Beutler JJ, Mali WP; SMART Study Group: Influence of atherosclerosis on age-related changes in renal size and function. Eur J Clin Invest. 2003;33(1):34–40. doi: 10.1046/j.1365-2362.2003.01091.x 12492450

[12] Chronic Kidney Disease Prognosis Consortium, Matsushita K, van der Velde M, Astor BC, Woodward M, Levey AS, de Jong PE, et al.: Association of estimated glomerular filtration rate and albuminuria with all-cause and cardiovascular mortality in general population cohorts: a collaborative meta-analysis. Lancet. 2010;375(9731):2073–81. doi: 10.1016/S0140-6736(10)60674-5 20483451 PMC3993088

[13] Kumar P, Shen Q, Pivetti CD, Lee ES, Wu MH, Yuan SY. Molecular mechanisms of endothelial hyperpermeability: implications in inflammation. Expert Rev Mol Med. 2009;11:e19. doi: 10.1017/S1462399409001112 19563700 PMC2828491

[14] Umanath K, Lewis JB. Update on Diabetic Nephropathy: Core Curriculum 2018. Am J Kidney Dis. 2018;71(6):884–895. doi: 10.1053/j.ajkd.2017.10.026 29398179

[15] Ohkubo Y, Kishikawa H, Araki E, Miyata T, Isami S, Motoyoshi S, et al.: Intensive insulin therapy prevents the progression of diabetic microvascular complications in Japanese patients with non-insulin-dependent diabetes mellitus: a randomized prospective 6-year study. Diabetes Res Clin Pract. 1995;28(2):103–17. doi: 10.1016/0168-8227(95)01064-k 7587918

[16] Intensive blood-glucose control with sulphonylureas or insulin compared with conventional treatment and risk of complications in patients with type 2 diabetes (UKPDS 33). UK Prospective Diabetes Study (UKPDS) Group. Lancet. 1998;352(9131):837–53. 9742976

[17] ADVANCE Collaborative Group, Patel A, MacMahon S, Chalmers J, Neal B, Billot L, Woodward M, et al.: Intensive blood glucose control and vascular outcomes in patients with type 2 diabetes. N Engl J Med. 2008;358(24):2560–72. doi: 10.1056/NEJMoa0802987 18539916

[18] Action to Control Cardiovascular Risk in Diabetes Study Group, Gerstein HC, Miller ME, Byington RP, Goff DC Jr, Bigger JT, Buse JB, et al.: Effects of intensive glucose lowering in type 2 diabetes. N Engl J Med. 2008;358(24):2545–59. doi: 10.1056/NEJMoa0802743 18539917 PMC4551392

[19] Gedebjerg A, Almdal TP, Berencsi K, Rungby J, Nielsen JS, Witte DR, et al.: Prevalence of micro- and macrovascular diabetes complications at time of type 2 diabetes diagnosis and associated clinical characteristics: A cross-sectional baseline study of 6958 patients in the Danish DD2 cohort. J Diabetes Complications. 2018;32(1):34–40. doi: 10.1016/j.jdiacomp.2017.09.010 29107454

[20] Nakamura M, Kitamura H, Konishi S, Nishimura M, Ono J, Ina K, et al.: The endocrine pancreas of spontaneously diabetic db/db mice: microangiopathy as revealed by transmission electron microscopy. Diabetes Res Clin Pract. 1995;30(2):89–100. doi: 10.1016/0168-8227(95)01155-2 8833629

[21] Parving HH, Viberti GC, Keen H, Christiansen JS, Lassen NA: Hemodynamic factors in the genesis of diabetic microangiopathy. Metabolism. 1983;32(9):943–9. doi: 10.1016/0026-0495(83)90210-x 6350816

[22] Nakayama H, Kato T, Nakayama S, Kaku H, Muraishi K, Tokubuchi I, et al.: Cross-sectional and Longitudinal Analyses of Factors Contributing to the Progressive Loss of the β-cell Function in Type 2 Diabetes. Intern Med. 2015;54(16):1971–6. doi: 10.2169/internalmedicine.54.4351 26278286

[23] Wang JC, Bennett M: Aging and atherosclerosis: mechanisms, functional consequences, and potential therapeutics for cellular senescence. Circ Res. 2012;111(2):245–59. doi: 10.1161/CIRCRESAHA.111.261388 22773427

[24] Man JJ, Beckman JA, Jaffe IZ: Sex as a Biological Variable in Atherosclerosis. Circ Res. 2020;126(9):1297–1319. doi: 10.1161/CIRCRESAHA.120.315930 32324497 PMC7185045

[25] Hemmelgarn BR, Manns BJ, Lloyd A, James MT, Klarenbach S, Quinn RR, et al.; Alberta Kidney Disease Network. Relation between kidney function, proteinuria, and adverse outcomes. JAMA. 2010;303(5):423–9. doi: 10.1001/jama.2010.39 20124537

[26] Go AS, Chertow GM, Fan D, McCulloch CE, Hsu CY: Chronic kidney disease and the risks of death, cardiovascular events, and hospitalization. N Engl J Med. 2004;351(13):1296–305. doi: 10.1056/NEJMoa041031 15385656

[27] Gerstein HC, Mann JF, Yi Q, Zinman B, Dinneen SF, Hoogwerf B, et al.; HOPE Study Investigators. Albuminuria and risk of cardiovascular events, death, and heart failure in diabetic and nondiabetic individuals. JAMA. 2001;286(4):421–6. doi: 10.1001/jama.286.4.421 11466120

[28] Jansen MA, Hart AA, Korevaar JC, Dekker FW, Boeschoten EW, Krediet RT; NECOSAD Study Group. Predictors of the rate of decline of residual renal function in incident dialysis patients. Kidney Int. 2002;62(3):1046–53. doi: 10.1046/j.1523-1755.2002.00505.x 12164889

[29] Kovesdy CP, Lott EH, Lu JL, Malakauskas SM, Ma JZ, Molnar MZ, et al.: Outcomes associated with microalbuminuria: effect modification by chronic kidney disease. J Am Coll Cardiol. 2013;61(15):1626–33. doi: 10.1016/j.jacc.2012.11.071 23500283 PMC3625505

[30] Hishida M, Shafi T, Appel LJ, Maruyama S, Inaguma D, Matsushita K: Lower levels of proteinuria are associated with elevated mortality in incident dialysis patients. PLoS One. 2019;14(12):e0226866. doi: 10.1371/journal.pone.0226866 31869391 PMC6927646

[31] Holman RR, Paul SK, Bethel MA, Matthews DR, Neil HA: 10-year follow-up of intensive glucose control in type 2 diabetes. N Engl J Med. 2008;359(15):1577–89. doi: 10.1056/NEJMoa0806470 18784090

[32] Shoji T, Masakane I, Watanabe Y, Iseki K, Tsubakihara Y: Committee of Renal Data Registry, Japanese Society for Dialysis Therapy. Elevated non-high-density lipoprotein cholesterol (non-HDL-C) predicts atherosclerotic cardiovascular events in hemodialysis patients. Clin J Am Soc Nephrol. 2011;6(5):1112–20. doi: 10.2215/CJN.09961110 21511840 PMC3087778

