# Supplementary material for: Low-grade proteinuria and atherosclerotic cardiovascular disease: A transition study of patients with diabetic kidney disease
Source: PLoS One. 2022 Feb 25;17(2):e0264568. doi: 10.1371/journal.pone.0264568 (PMC8880428; doi:10.1371/journal.pone.0264568)
Supplement: S1 Table — Abbreviations: eGFR, estimated glomerular filtration rate; HbA1c, glycated hemoglobin A1c; LDL, low density lipoprotein; OH-R, overhydration rate; RAAS, renin-angiotensin-aldosterone system; UPCR, urinary protein creatinine ratio. (PDF) [file pone.0264568.s001.pdf]

Supplementary table1. Characteristics of patients with or without the data of UPCR

Variables	UPCR		P value
	Not missing N=395	Missing N=48	
Age (years)	66.0 (12.8)	64.4 (12.3)	0.43
Male (%)	72.4	72.9	1.00
Classification of diabetes			
Type 1 (%)	3.3	6.3	0.40
Type 2 (%)	96.5	93.8	0.41
Pancreatic (%)	0.3	0.0	1.00
Diabetes duration (years)	15.3 [8,3-23.4]	18.3 [11.2-26.5]	0.09
Systolic blood pressure (mmHg)	152 (25)	152 (27)	0.98
eGFR (mL/min/1.73m^2)	5.6 (2.1)	5.2 (1.8)	0.37
Renal length (mm)	92.3 (13.9)	97.3 (13.6)	0.97
Body mass index			
At dialysis initiation	25.1 (4.6)	23.6 (4.4)	0.04
At discharge	22.6 (3.9)	21.2 (3.8)	0.02
Overhydration rate (%)	8.4 [4.1-16.0]	8.5 [4.0-13.4]	0.73
Laboratory data			
Albumin (g/dL)	2.9 (0.6)	2.9 (0.5)	0.74
LDL (mg/dL)	92 (37)	87 (31)	0.40
HbA1c (%)	6.0 (0.8)	6.2 (0.9)	0.25
Prior history (%)			
Advanced diabetic retinopathy	43.2	46.3	0.74
Coronary artery disease	17.0	16.7	1.00
Heart failure	22.5	25.0	0.72
Cerebral infarction	13.2	14.6	0.82
Peripheral arterial disease	11.9	20.8	0.11
Smoking status (%)			
Current	21.0	16.7	0.57
Past	26.6	27.1	1.00
Never	48.9	50.0	1.00
Unknown	0.4	0.6	0.42
Prescriptions			
Aspirin	38.5	41.7	0.75

Statin	43.5	31.3	0.12
Insulin	36.7	35.4	1.00
RAAS inhibitors	54.4	68.8	0.07

Abbreviations: eGFR, estimated glomerular filtration rate; HbA1c, glycated hemoglobin A1c; LDL, low density lipoprotein; OH-R, overhydration rate; RAAS, renin-angiotensin-aldosterone system; UPCR, urinary protein creatinine ratio.
